# Exploring Potential Mechanisms Accounting for Iron Accumulation in the Central Nervous System of Patients with Alzheimer’s Disease

**DOI:** 10.3390/cells13080689

**Published:** 2024-04-16

**Authors:** Steven M. LeVine

**Affiliations:** Department of Cell Biology and Physiology, University of Kansas Medical Center, 3901 Rainbow Blvd., Mail Stop 3043, Kansas City, KS 66160, USA; slevine@kumc.edu

**Keywords:** Alzheimer’s disease, axon, demyelination, diabetes, glutamate, habenula, inflammation, insulin resistance, iron, lysosome, metabolic, microglia, mitochondria, MRI, traumatic brain injury

## Abstract

Elevated levels of iron occur in both cortical and subcortical regions of the CNS in patients with Alzheimer’s disease. This accumulation is present early in the disease process as well as in more advanced stages. The factors potentially accounting for this increase are numerous, including: (1) Cells increase their uptake of iron and reduce their export of iron, as iron becomes sequestered (trapped within the lysosome, bound to amyloid β or tau, etc.); (2) metabolic disturbances, such as insulin resistance and mitochondrial dysfunction, disrupt cellular iron homeostasis; (3) inflammation, glutamate excitotoxicity, or other pathological disturbances (loss of neuronal interconnections, soluble amyloid β, etc.) trigger cells to acquire iron; and (4) following neurodegeneration, iron becomes trapped within microglia. Some of these mechanisms are also present in other neurological disorders and can also begin early in the disease course, indicating that iron accumulation is a relatively common event in neurological conditions. In response to pathogenic processes, the directed cellular efforts that contribute to iron buildup reflect the importance of correcting a functional iron deficiency to support essential biochemical processes. In other words, cells prioritize correcting an insufficiency of available iron while tolerating deposited iron. An analysis of the mechanisms accounting for iron accumulation in Alzheimer’s disease, and in other relevant neurological conditions, is put forward.

## 1. Introduction

A common pathological feature observed among numerous neurological disorders is an elevated level of iron within the central nervous system (CNS). This increase in iron occurs within cortical and/or subcortical CNS regions and has been observed in Alzheimer’s disease, amyotrophic lateral sclerosis, multiple sclerosis, Parkinson’s disease, and numerous other neurological diseases [1,2]. Iron is necessary for all living cells and is used for a wide range of biochemical reactions including many with enrichment within the nervous system, e.g., neurotransmitter metabolism, myelination, and mitochondrial complex activity [3,4]. Common themes for CNS iron accumulation may be the need to acquire extra iron to make up for a functional iron deficiency in order to maintain support for essential biochemical reactions, or to address an increased requirement for iron, e.g., due to an upregulation or alteration of metabolic processes. The former mechanism, a functional iron deficiency, has been postulated to occur in Alzheimer’s disease as well as some other neurological conditions [4,5,6], while the latter mechanism may occur as a corollary to inflammation, development, insulin resistance, or diabetes. Thus, the reasons leading to elevated levels of CNS iron may vary.

Iron has been directly or indirectly implicated in disease processes. Ascertaining whether a functional iron deficiency advances disease, or the accumulated iron has detrimental effects, will allow a greater understanding of pathogenic mechanisms. Fundamental to this understanding is addressing the reasons why iron accumulates. Insights about the causes of increased iron levels in CNS diseases can be ascertained by considering the timing of iron accumulation relative to the development of various pathological changes, as well as by examining the mechanisms of iron accumulation occurring in other neurological disorders. 

## 2. Pathogenic Processes Leading to a Functional Iron Deficiency 

**Figure 1:**
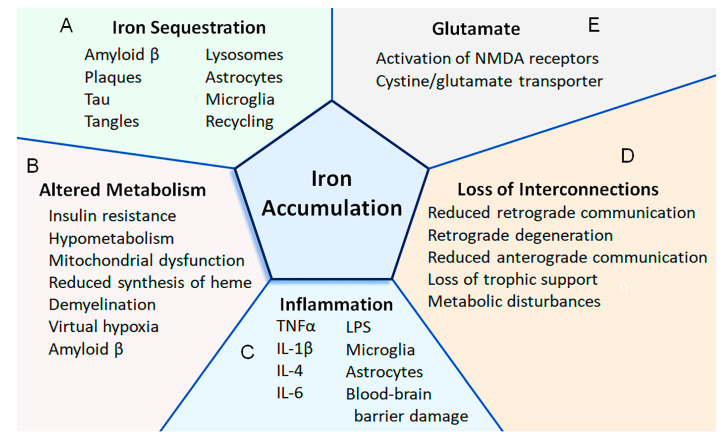
Putative mechanisms that lead to the accumulation of iron in the CNS of patients with Alzheimer’s disease. (**A**) Iron can become sequestered by being bound to amyloid or tau, accumulating within plaques and tangles, and retained within lysosomes, astrocytes, microglia, and structures (e.g., mitochondria, ferritin) that do not undergo proper recycling (Section 2). (**B**) Altered metabolism can increase the uptake of iron. For instance, as the demand for energy increases (e.g., due to demyelination causing virtual hypoxia), the energy requirements increase (e.g., to pump additional ions across the axolemma). An increased uptake of iron can also be in response to insulin resistance and mitochondrial dysfunction. Amyloid β can also activate cells to increase the expression of proteins involved with iron transport and potentially increase the uptake of iron (Section 3, Section 5 and Section 7). (**C**) Increased iron uptake can be due to inflammation. Various cytokines (i.e., IL-1β, IL-4, IL-6, and TNFα) cause the upregulation of proteins involved with iron uptake. Some cytokines can also reduce the expression of ferroportin (i.e., indirectly via upregulation of hepcidin), which is involved with iron export. Lipopolysaccharides can also increase or decrease the expression of proteins involved with iron uptake or export, respectively. Astrocytes can become activated (e.g., by IL-6) to secrete hepcidin, while other cytokines increase the uptake of iron within the mitochondria of astrocytes. Damage to the blood–brain barrier can cause extravasation of red blood cells and iron-containing proteins. Activated microglia can phagocytose debris containing iron, and this iron may become unavailable for use (Section 5). (**D**) As neurons degenerate, interconnections are lost between CNS regions. When interconnections are lost in other neurological conditions (e.g., traumatic brain injury, axotomy), iron can become deposited in CNS regions (e.g., thalamus), and the expression of proteins involved with iron uptake or iron export can be increased or decreased, respectively. Loss of interconnections reduces both retrograde and anterograde communications and causes loss of trophic support, metabolic disturbances, and neurodegeneration (Section 10). (**E**) The cystine/glutamate transporter may be increased in Alzheimer’s disease, and glutamate may play a role in increasing the uptake of iron. For instance, activation of the NMDA receptor can increase both the uptake of iron and the expression of proteins involved with iron uptake (Section 6).

A functional iron deficiency can lead cells to perceive a low iron status, thereby causing compensatory mechanisms [6]. Iron can become sequestered and unavailable for use by multiple mechanisms resulting in a functional iron deficiency. Besides becoming trapped in protein aggregates (e.g., amyloid β, tau, α synuclein), iron can become unavailable by processing defects (e.g., impaired delivery of iron from the lysosome to the cytosol or mitochondria), diminished recycling (e.g., disrupted mitophagy), decreased production or altered metabolism of heme and iron-containing proteins, etc. [4,6,7,8] (Figure 1). In addition, as a CNS disease advances, iron from degenerating cells can become sequestered within reactive microglia [9,10,11], which accumulate ferritin [12,13,14]. Once iron becomes unavailable, cells experience a functional iron deficient state that has similarities with anemia [5]. Cells can respond to a functional deficiency of iron by taking up more iron and exporting less iron [4,6]. Thus, the accumulation of iron is consistent with a functional iron deficiency. 

Lysosomal acidification is necessary for the proper delivery of iron from the lysosome to the cytosol and mitochondria [8]. In Alzheimer’s disease, this process can become impaired due to disruptions in the assembly and function of v-ATPase, i.e., by amyloid precursor protein c terminal fragment or by mutations in presenilin 1, resulting in an elevated lysosomal pH [6,15,16,17,18,19]. In addition, cultured astrocytes expressing the ε4 allele of *APOE*, which increases the risk of sporadic Alzheimer’s disease, resulted in an increased lysosomal pH and a decreased endosomal pH, while the cytoplasmic pH remained unchanged, compared to astrocytes with the ε3 allele [20]. 

Besides becoming sequestered within lysosomes, iron binds to amyloid β and other protein aggregates, e.g., α synuclein, tau [6]. As more amyloid β becomes deposited, more iron can become sequestered (Figure 1A). Elevated amyloid β production can be a consequence of altered proteolytic digestion of amyloid precursor protein in response to a variety of stresses (e.g., inflammation, oxidative stress, and lower levels of oxygen, 2-oxoglutarate, or iron) or mutations in genes responsible for familial Alzheimer’s disease (e.g., *APP*, *PSN1*, and *PSN2*) [6,21,22,23]. Additionally, overall levels of amyloid β can increase if its clearance is decreased [23,24]. Thus, iron can become unavailable by mechanisms occurring during early phases of the disease (e.g., due to sequestration within lysosomes, binding to protein aggregates of amyloid β or tau accumulation) as well as by additional mechanisms as the disease advances (e.g., due to sequestration of iron within microglia). 

## 3. Processes Accounting for Increased Iron Accumulation

During Alzheimer’s disease, cells respond to pathological conditions by increasing their acquisition of iron. Recently, it was shown that amyloid β lowers iron levels in conditioned media from iPSC-derived astrocytes, which may have been due to greater iron uptake by these cells and an elevation in their mitochondrial activity [25]. Furthermore, the conditioned media had an increased percentage of apo-transferrin, which stimulated endothelial cells to increase iron transport [25]. In support of this, the expression of transcripts for transferrin was upregulated in the olfactory bulb of patients with early Alzheimer’s disease compared to control subjects [5]. Amyloid β was also found to increase the expression of divalent metal transporter 1 (DMT1), which is thought to mediate an increased uptake of non-transferrin-bound iron in immortalized microglia [26] (Figure 1B).

Ceruloplasmin is a ferroxidase that together with transferrin is thought to facilitate the uptake of iron [27]. The expression of the transcript for ceruloplasmin was upregulated in the olfactory bulb across all stages of Alzheimer’s disease [5] as well as being increased at the protein level in the CNS of patients with Alzheimer’s disease [28]. Notably, it has been proposed that for aceruloplasminemia, astrocytes cannot export iron resulting in its accumulation within astrocytes, and the diminished transfer of iron to neurons causes an iron insufficiency that contributes to neurodegeneration; this iron deficiency may occur early in the disease course, after which iron-mediated oxidative damage from astrocytes may be a factor [29,30,31].

Other data further support an increased effort to acquire iron during Alzheimer’s disease. An analysis of the Mayo Clinic RNA-seq dataset (syn5550404, Synapse.org; https://www.synapse.org/#!Synapse:syn5550404), which compared 134 patients with Alzheimer’s disease and 130 control subjects, revealed a significant increase in transcripts for the transferrin receptor (*TFRC*) in the temporal cortex of patients with Alzheimer’s disease [32]. In addition, there was a significant decrease in transcripts for ferritin light chain (*FTL*) and ferritin heavy chain (*FTH1*) in the cerebellum of patients with Alzheimer’s disease, which also had a significant reduction in ferroportin [32]. These changes are in line with expected changes due to a perceived iron-deficient state, i.e., the increase in transcripts for *TFRC* would facilitate more iron uptake, while the decreases in *FTL* and *FTH1* reflect that there is less available iron to put into storage. 

In line with the effort to acquire more iron, the export of iron is diminished in the CNS of patients with Alzheimer’s disease. Ferroportin functions as an iron exporter at the cellular level [33]. The transcripts for ferroportin were downregulated in patients with initial Alzheimer’s disease [5]. Hepcidin affects the availability of ferroportin, by stimulating its internalization and degradation [33,34]. At the protein level, ferroportin was reduced in a mouse model of Alzheimer’s disease and in the CNS of patients with Alzheimer’s disease [35,36,37].

## 4. Timing of Iron Accumulation in Alzheimer’s Disease

Increased iron accumulation can occur across all phases of a disease. Studying iron accumulation early in the course of disease has the advantage of having fewer and less pronounced associated pathological changes that occur during advanced disease stages (e.g., extensive astrocyte gliosis, microgliosis, neurodegeneration, and atrophy) that can make the interpretation of data more complex. As the disease progresses, the causes accounting for the accumulation of iron likely change, i.e., as a result of secondary pathological events such as inflammation and metabolic disturbances. In addition, the roles that different cell types perform in relation to iron accumulation may also change with the advancement of disease [31].

Let us examine the development of iron accumulation in Alzheimer’s disease, which can highlight different underlying mechanisms. Enhanced iron deposition is present in both Alzheimer’s disease and mild cognitive impairment, which is often considered a precursor of Alzheimer’s disease [38,39,40,41]. In addition, higher R2* values (indicative of iron accumulation) were observed in the basal ganglia of cognitively normal subjects with cerebrospinal fluid (CSF) biomarkers of Alzheimer’s disease compared to cognitively normal subjects without these biomarkers [42]. This presentation during early disease activity might suggest that iron accumulation occurs prior to neurodegeneration. Indeed, iron accumulation was observed within neurons, i.e., in association with tangles, in CNS tissue samples from both patients with Alzheimer’s disease and from patients with mild cognitive impairment [38,43,44]. Furthermore, as indicated above, many of the changes at the transcription level favor iron uptake early in the course of disease [5]. However, besides being a feature of established Alzheimer’s disease, neurodegeneration can occur in mild cognitive impairment, which can include other neuropathological changes, such as plaques, tangles, and astrocyte gliosis [45,46]. In addition to accumulating within neurons, increased levels of iron were observed within astrocytes and plaques in mild cognitive impairment [38]. As the disease advances, neurons degenerate and iron accumulates within microglia (discussed below).

## 5. Inflammation and Iron Accumulation

The mechanisms accounting for iron accumulation occurring early in the disease course may persist, but as the disease advances, additional processes come into play. Accumulation of iron within microglia is present late in the disease, but less is known about this phenomenon in preclinical disease stages [47,48,49]. Microglia becoming enriched with iron is possibly due to infiltrating microglia phagocytosing iron-containing debris following neurodegeneration, which is accompanied by the increased expression of ferritin, and downregulation of homeostatic markers, as microglia become activated [30,49,50]. In fact, ferritin accumulates in microglia in Alzheimer’s disease and in its animal models [12,13,14,51]. Activated, iron-enriched microglia may further pathogenesis, e.g., by iron-mediated oxidative damage or inflammasome activation, particularly in the presence of amyloid β [49]. 

The blood–brain barrier can become damaged in Alzheimer’s disease, which can result in extravasation of iron-containing proteins and even some red blood cells [52]. This in turn may activate the innate immune response to potentially limit infection [52]. The sequestration of iron by microglia may function to reduce its availability to bacteria, and together with microglial activation resulting in the secretion of various mediators, help to limit infection [9]. Inflammation can also reduce the uptake of iron in the brain, which may decrease iron availability, or cause cellular mis-localization of iron, such as the accumulation of iron within mitochondria, triggering iron-related toxicity (e.g., oxidative tissue damage) [9,10]. 

It has been proposed that neuronal iron accumulation is increased once microglia become activated (e.g., due to lipopolysaccharide), and their release of IL-6 stimulates astrocytes to secrete hepcidin [53] (Figure 1C). Hepcidin then downregulates ferroportin expression by neurons to reduce iron export resulting in the accumulation of iron in neurons, which leads to their death via apoptosis [53]. Alternatively, the downregulation of ferroportin can be explained as neurons limiting their loss of iron since they are experiencing a functional iron deficiency [4,5,6]. Lipopolysaccharide also increased the expression of DMT1 and the acquisition of non-transferrin-bound iron in cultured immortalized microglial cells, while the anti-inflammatory cytokine IL-4 increased the expression of the transferrin receptor and the uptake of transferrin-bound iron [26]. 

In ventral mesencephalic primary neuronal cultures, both IL-1β and TNFα induced the expression of the transferrin receptor and DMT1, which are involved with iron uptake and transport, and these cytokines were associated with a greater influx and decreased efflux of iron [54]. Furthermore, IL-1β and TNFα increased the uptake of iron within the mitochondria of primary cultured rat astrocytes [55]. In rat primary hippocampal neuronal cultures, IL-6 and TNFα increased the uptake of iron, and this was associated with increased expression of DMT1 and decreased expression of ferroportin, which is used for cellular iron export [56,57]. Similar responses were observed in cortical microglial cultures; also, hepcidin levels were increased in astrocyte and microglial cultures, which may facilitate some of these changes that lead to the accumulation of iron [56]. Transferrin receptor expression and iron uptake were also observed in epiplexus cells of the choroid plexus following intraperitoneal injections of lipopolysaccharide or interferon γ [58].

In the autoimmune disease multiple sclerosis, or in its animal model experimental autoimmune encephalomyelitis, numerous cytokines (including IL-1β, IL-6, and TNFα) are elevated [59,60]. This raises the possibility that these cytokines are influencing the uptake of iron in this disease by mechanisms similar to those described above [61,62]. Iron accumulation in CNS structures begins early in the disease course of multiple sclerosis. For instance, elevated iron levels are present in clinically isolated syndrome, which is considered a precursor of multiple sclerosis [63,64], and as discussed previously, iron deposition also occurs early in the course of Alzheimer’s disease, i.e., in mild cognitive impairment or in subjects with CSF biomarkers of Alzheimer’s disease [38,39,40,41,42]. In both clinically isolated syndrome and early multiple sclerosis, structural changes to the brain are due to axonal transection, demyelination, blood–brain barrier disruption, inflammatory cell infiltrates, etc. [65,66]. 

Besides inflammation, other mechanisms can contribute to an increased accumulation of iron in multiple sclerosis, which may have cross-relevance for Alzheimer’s disease. Axonal transection, or loss of neuronal interconnections, can lead to mechanisms accounting for iron accumulation (Figure 1D), as discussed below (Section 10. Reduced Connectivity and Iron Accumulation). In addition, various forms of stress, such as oxidative damage or inflammation, can cause iron dyshomeostasis and increased uptake of iron by mitochondria [55,67,68]. Demyelination may account for the elevated levels of iron in deep gray matter structures in multiple sclerosis by increasing the energy demand due to a greater expenditure needed to pump ions across the axolemma of demyelinated axons, which has been referred to as virtual hypoxia [62,69,70]. Elevated iron levels are also necessary during both myelination and remyelination [70,71,72], and upregulation of the transferrin receptor and elevated levels of iron observed in the marmoset model of multiple sclerosis have been postulated to be in support of remyelination efforts [73]. Of note, demyelination and axonal damage are relevant pathological features of Alzheimer’s disease [74], as are attempts at remyelination [75,76]: This suggests that some mechanisms of iron accumulation in multiple sclerosis overlap with those of Alzheimer’s disease.

## 6. Glutamate and Iron Accumulation

Glutamate excitotoxicity is a pathogenic mechanism of Alzheimer’s disease, particularly as the disease advances [77]. Overactivation of extrasynaptic N-methyl-D-aspartate (NMDA) receptors favors neuronal death while activation of synaptic NMDA receptors is important for neuronal survival [77]. In primary cultured neurons, activation of the NMDA receptors was found to increase the uptake of iron by both transferrin-mediated and non-transferrin-bound iron pathways [78]. In rat spinal cord explants, glutamate excitotoxicity (mediated by threohydorxyaspartate) increased expression of the iron transport proteins, transferrin receptor and DMT1, and increased the accumulation of iron [79]. In PC12 cells, treatment with glutamate increased the uptake of iron, which was accompanied by an increase in DMT1 expression, and one day following intracerebroventricular injection of glutamate in the rat, the iron content of the corpus callosum, hippocampus, and cerebellum was increased and was associated with an increase in the expression of DMT1 in the latter two regions, suggesting a non-transferrin-bound iron uptake mechanism [80] (Figure 1E).

Compared to cognitively normal subjects, immunoblotting of the medial temporal cortex of patients with Alzheimer’s disease revealed increases in the expression of the cystine/glutamate transporter, ceruloplasmin, ferritin light chain, and ferritin heavy chain, and trends for decreases of ferroportin and DMT1, but no increase in the amount of elemental iron [81]. There was also an increase in the glutamate-to-GABA ratio, but this was mostly driven by a decrease in GABA [81]. In addition, there was a significant positive correlation of the transferrin receptor with iron levels in patients with Alzheimer’s disease, but not in cognitively normal individuals [81], possibly indicating a response to sequestered iron in the former group. Together, these results support the role of glutamate in the uptake of iron.

## 7. Insulin Resistance and CNS Iron Accumulation

Iron accumulation in the CNS has been linked with insulin resistance and reduced cognitive performance in obese individuals [82] as well as with diabetes mellitus, aging, and Alzheimer’s disease [83]. Some have suggested that insulin resistance increases iron levels in the CNS which then facilitates the development of Alzheimer’s disease [84], while others have proposed that iron accumulation facilitates insulin resistance and hyperphosphorylation of tau [85]. In cultured adipose cells, insulin was found to increase transferrin receptor expression at the cellular surface and increase the uptake of iron [86,87]. Besides affecting iron uptake, insulin resistance is thought to advance multiple other facets of Alzheimer’s disease pathogenesis including metabolic and mitochondrial dysfunction [88,89]. 

In both mild cognitive impairment and Alzheimer’s disease, hypometabolism is seen in numerous CNS structures [90]. Some CNS regions with hypometabolism in patients with Alzheimer’s disease also displayed iron accumulation (temporal cortex, parietal cortex, and hippocampus), but this association was not observed in other structures that displayed only one of these features, i.e., hypometabolism (precuneus lobe, cingulate gyrus, occipital lobe) or iron accumulation (globus pallidus, caudate, putamen) [91]. It has been proposed that the binding of heme to amyloid β reduces heme’s availability, resulting in diminished mitochondrial function and compensatory mechanisms, including attempts at upregulation of heme synthesis and iron accumulation [7]. More work is necessary to establish links between insulin resistance, hypometabolism, and iron accumulation in Alzheimer’s disease.

## 8. Cortical vs. Subcortical Iron Accumulation

In conditions thought to lead to Alzheimer’s disease, and in the disease itself, iron accumulates in both cortical and subcortical regions. For instance, when comparing cognitively normal subjects with or without CSF biomarkers of Alzheimer’s disease, there were elevated levels of iron (R2* values) in the basal ganglia in the former group [42], and in a longitudinal cross-sectional study, iron levels (R2* values) in the basal ganglia of patients with Alzheimer’s disease increased with time [92]. 

Elevated levels of iron were also observed in cortical regions, e.g., the temporal lobe, of patients with Alzheimer’s disease and were associated with cognitive decline [92,93]. Furthermore, iron accumulation was modestly correlated with the amount of amyloid β accumulation in mild cognitive impairment and Alzheimer’s disease [39,93]. Besides amyloid β, iron accumulation has also been associated with the aggregation of tau and neurodegeneration in patients with Alzheimer’s disease, particularly within the inferior temporal gyrus, as measured by quantitative susceptibility mapping, Tau-PET, and structural MRI [94]. 

These findings raise the following questions: (1) Are the mechanisms of iron accumulation the same among CNS regions (e.g., cortical vs. deep gray matter structures), are they different between regions, or is there a mixture of mechanisms? (2) What is the interrelationship of iron accumulation between different cell types? (3) What is the connection between iron accumulation and neurodegeneration, and other pathological changes (e.g., amyloid β and tau aggregates); in other words, is the accumulation of iron an effect of other pathological changes, or is it the cause, or a mixture of cause and effect? To begin to address these questions, we can examine the mechanisms of iron accumulation in other neurological disorders and evaluate whether there are shared processes with Alzheimer’s disease. But first, let us examine one brain region, the habenula, that may have a particularly relevant role in iron uptake.

## 9. The Choroid Plexus, Habenula, and Iron Accumulation

**Figure 2:**
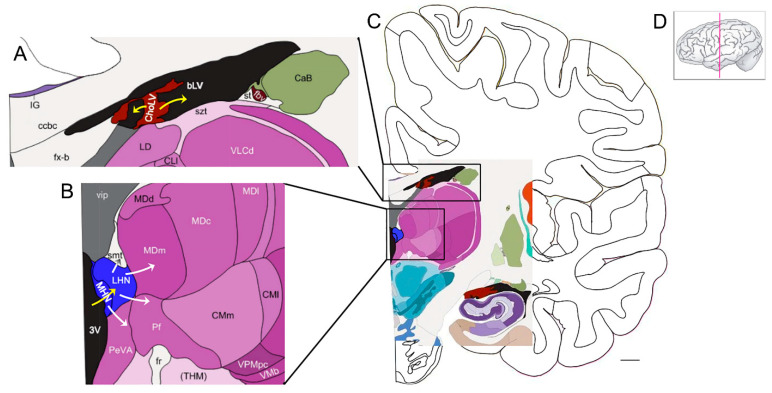
The habenula is a putative hub for the uptake of iron from the ventricular CSF and delivery of iron to subcortical structures (see Section 9). (**A**) Iron transport at the choroid plexus (brown)**:** Transferrin (with bound iron), delivered via fenestrated capillaries in the stroma, binds the transferrin receptor on epithelial cells of the choroid plexus and undergoes clathrin-mediated endocytosis. The export (yellow arrows) of ferrous iron into CSF of the ventricles (black) is mediated via ferroportin on epithelial cells. These cells also produce ceruloplasmin and transferrin. Ceruloplasmin converts ferrous iron to ferric iron, which then binds to transferrin. Note that other proteins involved with iron transport are also produced by the choroid plexus. (**B**) At the habenula (blue), the uptake of iron (yellow arrow) is via transferrin, in the ventricular CSF, binding to the transferrin receptor on habenular cells. Other routes for iron uptake by habenular cells may apply. The habenula exports iron via ferroportin for delivery to other CNS structures (white arrows), especially those with interconnections to the habenula. (**C**) A low-power view of the structures depicted in A and B. Note the choroid plexus (brown) adjacent to the hippocampus. (**D**) The pink line indicates the coronal plane of the section. Bar = ~4.4 mm. Abbreviations: bLV—body of lateral ventricle; CaB—body of caudate; ccbc—body of corpus callosum, caudal portion; ChoLV—choroid plexus of lateral ventricle; CLI—lateral division of central lateral nucleus; CMI—lateral division of centromedian nucleus of thalamus; CMm—medial division of centromedian nucleus of thalamus; fr—fasciculus retroflexus (habenuno-interpeduncular tract); fvb—blood vessels of forebrain; fx-b—body of the fornix; IG—indusium griseum; LD—lateral dorsal nucleus of thalamus; LHN—lateral habenular nucleus; MDc—parvocellular (central) division of MD; MDd—densocellular (paralamellar) division of MD (mediodorsal nucleus of thalamus); MDI—multiform (lateral) division of MD; MDm—magnocellular (medial) division of MD; MHN—medial habenular nucleus; 3V—third ventricle; PeVA—periventricular area of thalamus; Pf—parafascicular nucleus of thalamus; st—stria terminalis; szt—stratum zonale of thalamus; (THM)—thalamus; vip—velum interpositum; VLCd—dorsal subdivision of VLC (caudal division of ventral lateral nucleus of thalamus); VMb—basal ventral medial nucleus; VPMpc—parvocellular division of ventral posterior medial nucleus (VPM). This figure was generated from materials available from the Allen Institute for Brain Science at Interactive Atlas Viewer: Atlas Viewer (brain-map.org) [103].

The choroid plexus expresses numerous transcripts and proteins involved with iron transport, and it has been proposed to have a role in iron uptake followed by iron export into the ventricular CSF [95,96,97,98,99,100] (Figure 2A). The delivery of iron into the CSF by the choroid plexus is supported by its production of ferroportin, transferrin, and ceruloplasmin, and presumably the secretion of the latter two into the CSF [96,97,98,99,100]. Ferroportin is believed to export ferrous iron, while ceruloplasmin oxidizes it to the ferric form, which allows it to bind transferrin [101,102]. 

The habenula, which is adjacent to the third ventricle, may have a prominent role in iron uptake from the CSF (Figure 2B). The habenula has a high expression level of the transferrin receptor [104,105,106,107], and ferroportin is expressed in high levels within neuronal fibers and cell bodies at the medial habenula [99]. These results indicate that there is a lot of iron flux in this region [99], and this route could be relevant for iron delivery to subcortical structures, e.g., the thalamus (Figure 2B). Furthermore, regions with projections to the habenula may have elevated iron uptake [104]. Transferrin given via the peripheral blood or via intracerebroventricular injection was detected in the habenula and other regions of the CNS within hours after injection [108,109]. Besides the choroid plexus-to-CSF-to-habenula-to-thalamus (or other interrelated subcortical structure, see below) route, other mechanisms (e.g., uptake of transferrin across capillaries or delivery of transferrin from oligodendrocytes) are also likely substantially involved in the delivery of iron [106,110].

The habenula is connected with numerous structures, with outputs largely via the fasciculus retroflexus to the midbrain and inputs via the stria medullaris from the basal ganglia and limbic system [111,112]. Interconnected structures include the thalamus, putamen, head of the caudate, globus pallidus internus, rostromedial tegmental nucleus, posterior hippocampus, amygdalar nuclei, substantia nigra, interpeduncular nucleus, basal nucleus of Meynert, dorsal raphe nuclei, septal nuclei, and medial prefrontal cortex [111,112]. Many of these structures, i.e., those in the basal ganglia, have high levels of iron that tend to increase with age [113]. 

Iron levels are also present at relatively high levels in the habenula [114,115], and iron levels increase in the habenula during depression [116] and inflammation, i.e., in a cerebral model of experimental autoimmune encephalomyelitis, which is likely tied to the activation of hypoxia-inducible factor 1 [107,117,118]. Given these findings and the role of the habenula in iron uptake [106], it raises the prospect that this structure is facilitating some of the accumulation of iron that is observed in Alzheimer’s disease. Interestingly, in subjects with frontotemporal dementia, the habenula volume decreased, and in fact, it had the largest percentage decrease of all regions examined; but, in age-matched patients with early-onset Alzheimer’s disease, the volume remained unchanged compared to control subjects [119] suggesting that this putative mechanism for iron uptake can function while Alzheimer’s disease progresses.

## 10. Reduced Connectivity and Iron Accumulation

Studying traumatic brain injury may further our understanding of iron accumulation in Alzheimer’s disease. This may seem counterintuitive (for instance, due to edema and hemorrhage in the former which might confound the interpretation of iron deposition), but there are changes downstream from the impact that may overlap with some processes involved with iron accumulation in Alzheimer’s disease. From an investigative standpoint, traumatic brain injury has the advantage of having both a known starting point and site of primary injury, to some degree, which are useful references for subsequent analyses.

In humans with traumatic brain injury, as well as in animal models, impairment can occur in a range of cortical and subcortical structures, including the thalamus [120,121,122,123,124]. Changes to the thalamus, secondary to cortical injury, include neurodegeneration, inflammatory events such as microgliosis and astrocytosis, and iron accumulation [120,121,123,125,126]. Increased iron deposition in deep gray matter structures, i.e., globus pallidus and thalamus, even occurs in patients with mild traumatic brain injury (admittedly “mild” is a relative term since this injury can still cause appreciable changes) [127,128]. The accumulation of iron and persistence of other pathological changes in the thalamus do not appear to be a consequence of hemorrhage since there was no evidence of hemorrhage in the thalamus during acute or post-acute periods [120,121,129].

Within the thalamus, elevated levels of iron may arise from secondary responses that are multifactorial. These include responding to the loss of interconnections [130,131,132], which can affect both retrograde and anterograde communications and trigger both neuronal and glial responses [129,133] including microgliosis, astrocytosis, and neuronal changes (e.g., autophagy) that extend to neurodegeneration [120,121,123,134]. Changes within thalamic neurons are numerous and include reduced nuclear volume, decreased nuclear NeuN staining, shrunken size, swollen neuritic processes, increased expression of amyloid precursor protein within axons, loss of staining for MAP-2, Nissl dissolution, swollen or fragmented mitochondria, electron-dense structures, silver-stained damaged neuritic processes, etc. [129,133,135,136]. Furthermore, profiles of metabolites indicating alterations to energy metabolism and excitatory neurotransmission were observed in the thalamus following fluid percussion brain injury [137].

Some neurons will survive following the loss of interconnections, e.g., in the thalamus, due to traumatic brain injury [129,136], while others will undergo neurodegeneration that can extend over time, i.e., as a post-acute process [120,121,138,139]. If thalamic neurons survive the loss of interconnection with cortical structures, then they could be attempting to retain and increase their iron levels to accommodate an upregulation of metabolic changes in response to the injury. Reduced trophic support can follow the loss of interconnections and would cause cellular stress, e.g., mitochondrial impairment [140], which may trigger cells to respond by increasing iron uptake [62].

It is worth considering alterations to iron homeostatic processes within surviving neurons or within neurons that are undergoing changes that eventually lead to neurodegeneration. Unfortunately, there is insufficient data on the expression of proteins involved in iron homeostatic processes in the thalamus after traumatic brain injury. However, in the ipsilateral cortex of mice, traumatic brain injury led to iron accumulation, an increased expression of transferrin receptor (used for iron uptake), and decreased expression of ferroportin (used for iron export) one day after injury [141]. Similar responses for transferrin receptor 1 and ferroportin were seen in the rat cortex and hippocampus at 7 days post-injury [142], and transferrin expression was elevated in a coronal section of the brain, 4 mm thick, at 28 days [143]. In mice with traumatic brain injury, the expression of transferrin receptor 1, detected by immunofluorescence, was significantly elevated at 12 and 24 h, but not at 48 and 72 h, in neurons of the ipsilateral cortex [144]. Immunoblotting of the cortex at 12 h confirmed the elevation of transferrin receptor 1 and also showed an elevation of transferrin [144,145]. Although the timing can vary somewhat, the responses are occurring relatively close to the moment of primary injury, i.e., impact. In comparison, it is relevant to recognize that the development of pathology and the loss of substantial numbers of interconnections likely will occur more gradually in Alzheimer’s disease [146]. Since responses related to iron uptake take place over time in Alzheimer’s disease, only a relatively limited number of cells may be responding at a given point in time, e.g., at the time of tissue collection, and thus, may not be readily detected by immunoblotting or even immunofluorescence.

Following brain injury in mice, ceruloplasmin levels increased in the ipsilateral hemisphere at 6 and 24 h post-injury [147]. Ceruloplasmin is expressed by astrocytes [148], which is thought to work with transferrin to promote iron uptake [27]. Consistent with this view, ceruloplasmin-deficient mice had a decrease in total iron levels in the cortex and striatum [149]. In contrast, the absence of ceruloplasmin was thought to lead to iron accumulation in neurons following head injury [147], but it is possible that this latter study was detecting a redistribution of stainable iron, e.g., due to increased autophagy [150].

Following axotomy of the sciatic nerve in rats, increased levels of transferrin receptor were observed in Schwann cells, macrophages, and in regenerating neurites reaching a peak by 4 days after injury and declining thereafter [151]. The level of iron uptake was also dramatically increased following injury, which peaked at 3 days after injury, with the accumulated iron predominately at the crush injury site, and uptake declining thereafter [151]. Iron is used in support of the proliferation and maturation of Schwann cells, and iron uptake is required for myelination [152,153].

In a transgenic mouse model of amyotrophic lateral sclerosis, both transferrin receptor and iron levels were increased in the spinal cord compared to wild-type mice [154]. Similarly, in regenerating rat motor neurons, both transferrin receptor levels and iron uptake were increased [155]. Besides iron uptake via the transferrin receptor, transferrin can be taken up by terminals of motor neurons in a nonspecific manner after an intramuscular injection; the neurons then transport it retrogradely to the soma [156]. Intramuscular injections of iron and ferritin preparations were also found to undergo uptake and transport by associated nerves and elevated levels of iron persisted in Schwann cells [157].

Increased iron accumulation following traumatic brain injury was also associated with increased activity by the mitochondrial calcium uniporter [141]. The link between iron and the mitochondrial calcium uniporter may be relevant in other neurodegenerative conditions, including Alzheimer’s disease [158,159,160]. Intermittent theta burst stimulation, which may improve cognition in Alzheimer’s disease, was shown in APP/PS1 mice to increase the expression of iron–sulfur cluster assembly 1, which is needed for proper mitochondrial respiration [161].

Besides being present following traumatic brain injury [120,121,162,163], thalamic atrophy is present in Alzheimer’s disease [164], and impaired thalamic interconnections have been observed in both mild cognitive impairment and Alzheimer’s disease [165]. Furthermore, impaired functional connections, e.g., default mode network and executive function, are altered following both traumatic brain injury and early stages of Alzheimer’s disease [163,166,167]. Inflammation is also widespread in Alzheimer’s disease, including within the thalamus [168]. Inflammation (e.g., microgliosis) together with atrophy has also been seen in mouse models of Alzheimer’s disease [11,169]. Given that both Alzheimer’s disease and traumatic brain injury cause the loss of interconnection and inflammation in the thalamus, it is likely that some processes accounting for iron uptake in this structure are similar between these conditions. But as stated previously, the responses tied to iron uptake in Alzheimer’s disease would likely be operating at a lower and more sustained level compared to that following the sudden impact of traumatic brain injury, although associated post-acute changes would occur more slowly.

## 11. Conclusions

Iron has an essential role in numerous biochemical reactions, and cells have integrated processes designed to meet their requirements for iron [170]. The total level of iron within a tissue, however, does not necessarily reflect the amount of iron that is available for use [4,6]. This can potentially occur during normal aging [4], which results in an increase of iron within the brain [171]. In a disease state, where iron accumulates beyond that observed due to normal aging, the accumulated iron can become trapped within the lysosome, bound to protein aggregates, sequestered within microglia, etc., resulting in a functional deficiency of iron [4,6]. When iron is unavailable, cells can respond by increasing iron import and decreasing its export [4,6]. If the sequestered iron is combined with the extra iron needed to make up for a functional deficiency, then the total level of iron can increase while the amount of available iron for use is unchanged or decreases depending on whether compensatory mechanisms are sufficient [4,6]. Thus, a functional deficiency of iron is not mutually exclusive with iron accumulation [4,6]. Iron levels can also increase if metabolic processes are upregulated, thereby increasing the requirements for iron, or during inflammatory processes when iron is sequestered.

Although the mechanisms that have been discussed that are purported to lead to iron accumulation in Alzheimer’s disease are supported by a variety of scientific findings, the evidence for their relative contributions, and how they might change during the course of the disease, is limited. This limitation highlights the need for further studies, particularly those during the early stages of the disease. Iron deposition has been proposed to be a biomarker of disease activity in a variety of neurological disorders, including Alzheimer’s disease [42,172,173,174]. However, as the disease develops, the rate of iron accumulation may reflect the dynamics of various pathological features and change accordingly [4]. For instance, the accumulation of iron early in the disease course could be a response to a functional deficiency of iron, while its persistence in more advanced stages of disease, e.g., following neurodegeneration and atrophy, could represent its redistribution, at least in part, to microglia and astrocytes.

As iron builds up during a disease state, a question naturally arises: Is the accumulated iron deleterious? Although iron can catalyze damaging oxidative reactions, its sequestration, e.g., by amyloid or tau, may serve a protective function by limiting these reactions [175,176]. Other defense mechanisms, e.g., antioxidants and related enzymes, can also limit the damaging effects of iron-catalyzed reactions. However, if these get overwhelmed or depleted, then the accumulated iron has the potential to facilitate tissue damage [177].

In summary, pathological events in Alzheimer’s disease cause the sequestration of iron, and/or an elevated requirement for iron, leading to a perceived deficiency that triggers an increased uptake and retention of iron resulting in a net increase in accumulated iron.
